# The potential impact of the extended vitamin D fortification policy during pregnancy varies by continent of origin - a population-representative Swedish cohort

**DOI:** 10.1007/s00394-025-03584-2

**Published:** 2025-01-24

**Authors:** Mathilda Forsby, Anna Winkvist, Ciara Mooney, Frida Dangardt, Jenny M. Kindblom, Linnea Bärebring, Hanna Augustin

**Affiliations:** 1 Department of Internal Medicine and Clinical Nutrition, Institute of Medicine, University of Gothenburg, Gothenburg, Sweden; 2 Department of Molecular and Clinical Medicine, Institute of Medicine, University of Gothenburg, Gothenburg, Sweden; 3 Children’s Heart Center, The Queen Silvia Children’s Hospital, Sahlgrenska University Hospital, Gothenburg, Sweden; 4 Department of Drug Treatment, Sahlgrenska University Hospital, Gothenburg, Sweden

**Keywords:** Nutrients, Diet, Maternal health, Pregnant women, Food fortified, Dairy products.

## Abstract

**Purpose:**

We aimed to explore the potential impact of Sweden’s extended fortification policy, launched in 2018, on vitamin D intake during pregnancy, depending on continent of origin.

**Methods:**

The population-representative GraviD cohort was conducted within the antenatal care in 2013–2014 in Southwestern Sweden. Background data including country of origin were collected through questionnaires. In the third trimester, participants (*N* = 1761) answered a vitamin D questionnaire which included intakes of margarine, milk, and fermented milk. Reported vitamin D intake in 2013–2014 was compared to simulated vitamin D intake following the 2018 vitamin D fortification policy expansion.

**Results:**

Pre-expansion reported median intake of vitamin D from fortified foods differed by continent of origin (*p* < 0.001). Pre-expansion intake was highest among participants from Northern Europe (2.4 µg/day) compared to those from Continental Europe (2.0 µg/day, *p* = 0.002), Asia (1.6 µg/day, *p* < 0.001), and Africa (2.0 µg/day, *p* = 0.001). Post-expansion simulated median vitamin D intake from fortified foods was higher among participants from Northern Europe (6.3 µg/day) compared to Asia (5.0 µg/day, *p* < 0.001) and Africa (5.0 µg/day, *p* = 0.013). Participants from Continental Europe had the largest change (3.6 µg/day) between pre- and post-expansion, while those born in Asia had the smallest change (2.9 µg/day).

**Conclusion:**

The Swedish fortification policy expansion had a positive potential impact on vitamin D intake during pregnancy, but the effect depended on the continent of origin. The potential impact was smallest for participants from Asia and Africa, indicating that the current Swedish fortification policy is most beneficial for individuals of European origin.

**Supplementary Information:**

The online version contains supplementary material available at 10.1007/s00394-025-03584-2.

## Introduction

Vitamin D has an essential role in bone metabolism [1]. Severe vitamin D deficiency leads to inadequate bone mineralization, eventually resulting in rickets or osteomalacia [1]. Vitamin D deficiency has also been associated with extra-skeletal outcomes [2], including gestational complications such as preeclampsia [3], gestational diabetes [4], and impaired fetal growth [5]. Risk factors for vitamin D deficiency include older age [6], low sun-exposure [7], and low vitamin D intake [8].

Vitamin D consists of fat-soluble secosteroids which are present in both dietary and supplement sources [9]. Additionally, it is endogenously synthesized after exposure to solar ultraviolet-B radiation. In high latitude regions, such as the Nordic countries, the inadequate ultraviolet-B radiation during winter limits cutaneous vitamin D synthesis [10]. Thus, to maintain sufficient levels of vitamin D, often defined as 25-hydroxyvitamin D (25OHD) ≥ 50 nmol/L [9, 11], adequate dietary vitamin D intake is necessary, particularly in the Winter months [12]. According to the Nordic Nutrition Recommendations, the recommended intake of vitamin D is 10 µg/day for all adults, including pregnant individuals. However, for individuals with minimal or no sun exposure, the recommended intake is 20 µg/day [12]. Due to the limited number of foods naturally containing significant amounts of vitamin D [9], both voluntary and mandatory fortification policies have been implemented in several countries [13–18]. In Sweden, both margarine and milk have been mandatorily fortified since 2007 [19]. Initially, margarine was fortified with 7.5–10 µg vitamin D/100 g, and milk with ≤ 1.5% fat by weight was fortified with 0.38–0.5 µg/100 g. Despite this, dietary intake data in Sweden have shown inadequate dietary vitamin D intake. For example, the Riksmaten 2010-11 national survey revealed a median intake of 5.7 µg/day [20], similar to findings in Swedish studies of vitamin D intake during pregnancy [21, 22]. Consequently, an expanded fortification policy was introduced in 2018, to include a wider variety of foods and higher levels of vitamin D fortification [23, 24]. The mandatory level of vitamin D fortification was doubled in margarine (19.5–21 µg/100 g) and milk (0.95–1.10 µg/100 g). It was also extended to include milk with ≤ 3% fat by weight, as well as, plain and flavoured fermented milk with ≤ 3% fat by weight (0.75–1.10 µg/100 g). The expanded fortification policy also includes lactose free and plant-based milk and fermented milk alternatives [23, 24].

The expanded vitamin D fortification policy has not yet been thoroughly evaluated. Previous studies in the Nordic countries have however shown that individuals born in Asia and Africa are at a higher risk of insufficient vitamin D status than those of Nordic origin [8, 25–27]. Smaller studies have also found that vitamin D intake among individuals from Asia and Africa is lower [26, 28], which suggests that those with a greater need for higher intakes have the lowest reported intakes. This highlights the need to systematically examine dietary vitamin D intakes and evaluate current policies implemented to improve vitamin D intake and status, not just in the general population but particularly among high-risk groups. Therefore, the aim of this study was to explore the potential impact of the extended fortification policy in Sweden on vitamin D intake during pregnancy, depending on continent of origin.

## Method

This study is based on data from the population-representative GraviD cohort study, which was conducted in the south-west of Sweden during routine antenatal care visits between autumn 2013 and spring 2014. Recruitment took place at a total of 43 antenatal care units. All individuals whose pregnancy had not exceeded 16 gestational weeks were eligible for inclusion. Study information was provided in both verbal and written formats, in nine languages including Swedish, English, Arabic, French, Polish, Persian, Somali, Sorani, and Turkish. Written informed consent was obtained from all participants. The GraviD study was conducted in accordance with the Declaration of Helsinki and approved by the Regional Ethics Committee in Gothenburg, Sweden (protocol codes 2019–05219, 879 − 11, T439-13, T085-14).

In the first (gestational week < 17) and third (gestational week > 31) trimester of pregnancy, participants were requested to answer an analogue questionnaire including background information (i.e., education, origin, and eye color) and lifestyle factors (i.e., sun habits, and dietary and supplemental intake). The questionnaire was accessible in both Swedish and English. Interpreters were provided if needed in accordance with standard practices of antenatal care. Data of maternal age, parity, weight, height, and tobacco use were derived from medical records.

**Fig. 1:**
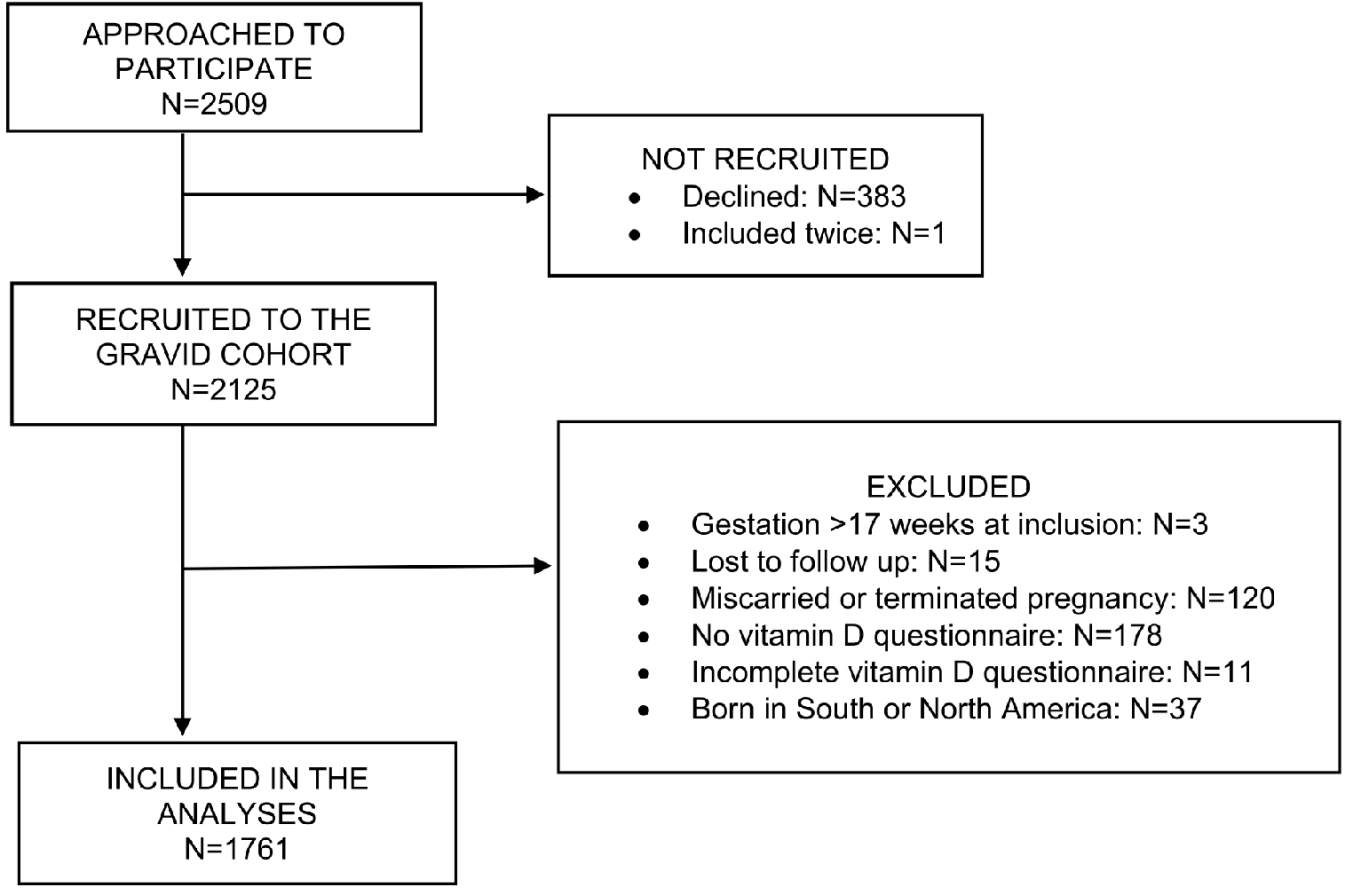
Flowchart of the GraviD cohort and included study participants in the analyses

Origin was defined by participants’ self-reported country of birth. Countries of birth were categorized to their continents and a total of four categories were used for the study (Northern Europe, Continental Europe, Asia, and Africa). Europe was divided by latitudes into Northern Europe (Nordic countries, Latvia, Lithuania, and the United Kingdom) and Continental Europe (rest of the continent Europe). Participants born in South or North America were considered insufficient in total number for further analyses and were therefore excluded (*N* = 37) (Fig. 1). None of the participants were born in Oceania. Participants included and excluded in the study are presented in Fig. 1.

### Dietary intake of vitamin D

In the third trimester, dietary intake of vitamin D reflecting the past two months was estimated using a short Vitamin D Questionnaire (VDQ). The VDQ has previously been validated against four-day food records and the biomarker 25OHD as reference methods [22]. In brief, the validation study showed that the estimated vitamin D intake reported by the VDQ significantly correlated with the intake reported through food records (rho = 0.51) and concentrations of 25OHD (rho = 0.162) [22]. The VDQ included four vitamin D-rich food sources: oily fish, margarine, milk, and fermented milk (i.e., yoghurt and sour milk). The participants were asked whether they consumed the food source (yes or no), and if so, they were further requested to specify the type of food, frequency of consumption, and quantity typically consumed (Supplementary material, figure S1).

As a first step, the VDQ in the third trimester in 2013–2014 (pre-expansion of the vitamin D fortification policy [19]) was used to estimate reported vitamin D intake pre-expansion. As a second step, a simulation of vitamin D intakes post-expansion was made, taking the expanded vitamin D fortification policy [23, 24] into account. The simulated vitamin D intake post-expansion was calculated using the food frequencies and amounts reported in the VDQ, adding the higher vitamin content in the fortified food introduced in 2018 in the vitamin D fortification policy expansion [23, 24]. Pre-expansion and post-expansion vitamin D content were derived from the National Food Agency’s database version 2013-10-04 and 2023-06-13, respectively. Table 1 provides the assumptions regarding the vitamin D content of vitamin D fortified foods included in the VDQ, both pre- and post-policy expansion. One serving of oily fish corresponded to 125 g and was assumed to contain 15.63 µg vitamin D. The assumed fish consumption was 2.5 servings per week or 3.5, 1.5, or 0.5 servings per month, depending on the answer (at least twice weekly, 3–4 times per month, 1–2 times per month, or more seldom). One serving of margarine as spread was assumed to be 8 g. Daily milk or fermented milk consumption corresponded to either 500, 200, or 50 g depending on the answer (more than 300 g/day, 100–300 g/day, or less than 100 g/day).

**Table 1:** Assumptions of vitamin D content of vitamin D fortified food included in the short vitamin D questionnaire, both pre- and post-expansion of the vitamin D fortification policy

	Vitamin D content/100 g	
Fortified food type	Pre-expansion^a^	Post-expansion^b^
Margarine	10 µg	20 µg
**Milk**		
Whole	0.02 µg	1 µg
Medium fat, low fat, and skimmed	0.5 µg	1 µg
Plant-based replacement with medium vitamin D content	0.75 µg	1 µg
Plant-based replacement with high vitamin D content	1.5 µg	1 µg
Organic plant-based replacement	0	1 µg
**Fermented milk**		
Plain	0.38 µg	1 µg
Flavoured	0 µg	1 µg
Greek or Turkish	0 µg	0 µg
Plant-based replacement	0 µg	1 µg

^a^ Pre-expansion refers to the vitamin D fortification policy prior to 2018 [19]

^b^ Post-expansion refers to the extended vitamin D fortification policy initiated in 2018 [23, 24]

Imputation was applied when type of food or quantity of consumption were missing. Missing values of milk type were replaced with mean vitamin D content of full fat and medium fat milk (1.8% of participants). Missing values of fermented milk type were replaced with mean vitamin D content of plain and flavoured fermented milk (2.5% of participants). When the quantity of consumption of milk or fermented milk was not recorded, the imputed value of a standard portion of 200 g/day from the National Food Agency’s database was used (0.8% of participants). Missing values for the frequency of fish intake were imputed using the corresponding recorded quantity of fish intake from the questionnaire completed in the first trimester (0.2% of participants). If more than one answer was recorded for type of food or quantity of consumption, the mean value of the recorded values was used (14% of participants). Missing data for both the question of food type and quantity of consumption were not imputed and, therefore, excluded from the analysis (0.6% of participants) (Fig. 1).

Values from fish, margarine, milk, and fermented milk were added together to calculate total intake of vitamin D from vitamin D-rich food per day. The maximum possible vitamin D intake from the VDQ according to these assumptions was 18.6 µg pre-expansion and 22.8 µg post-expansion.

### Statistical analyses

Descriptive statistics were presented as frequencies, percentages, medians and 25th and 75th percentiles, due to several of the variables not being normally distributed, visually assessed by histograms, boxplots, and quantile-quantile plots.

Difference in median vitamin D intake pre- and post-expansion was tested using Wilcoxon signed rank test. Kruskal Wallis test was conducted to analyse whether mean ranks of vitamin D intake from oily fish, margarine, milk, and fermented milk differed by continent of origin (Northern Europe, Continental Europe, Asia, and Africa). Post hoc analyses using Dunn’s test were conducted for pairwise comparisons, and adjustments for multiple tests were implemented using the Bonferroni correction.

IBM SPSS Statistics version 29.0 was used for all statistical analyses (IBM Corp., Armonk, NY, USA) and statistical significance was accepted at *p* < 0.05.

## Results

### Characteristics of study participants

The median age of the 1761 included participants was 31 years (Table 2). In the first trimester of pregnancy (gestational week ≤ 14), median BMI was 24 kg/m^2^ and 5% reported use of tobacco. The majority was born in Northern Europe (77%), followed by Asia (10%), Continental Europe (7%), and Africa (6%). Over half of the participants had studied at university level (60%), with the lowest proportion reported among those born in Africa (27%).

**Table 2:** Characteristics (median, 25th and 75th percentiles or percent) in the first trimester of pregnancy of all participants and stratified by continent of origin

	All participants (*N* = 1761)	Northern Europe (*N* = 1355)	Continental Europe (*N* = 131)	Asia (*N* = 173)	Africa (*N* = 102)
**Median (p25, p75)**					
**Age (years)**	31.3 (27.8, 34.6)	31.4 (28.1, 34.6)	30.6 (27.6, 34.3)	31.2 (27.5, 35.6)	29.0 (25.8, 33.9)
**Body mass index (kg/m** ^**2**^ **)**	23.6 (21.6, 26.3)	23.5 (21.5, 26.0)	23.4 (21.4, 26.0)	24.3 (21.9, 27.6)	24.5 (22.4, 28.2)
**N (%)**					
**Body mass index**					
< 18.5 kg/m^2^	33 (2)	23 (2)	2 (2)	7 (4)	1 (1)
25–30 kg/m^2^	441 (25)	327 (24)	33 (25)	47 (27)	34 (33)
> 30 kg/m^2^	173 (10)	123 (9)	12 (9)	24 (14)	14 (14)
**Education level**					
Primary	123 (7)	30 (2)	14 (11)	35 (21)	44 (46)
Secondary	578 (33)	450 (33)	44 (34)	58 (34)	26 (27)
University	1050 (60)	874 (65)	72 (55)	78 (46)	26 (27)
**Nulliparous**	721 (41)	583 (43)	52 (40)	60 (35)	26 (25)
**Tobacco use**	83 (5)	66 (5)	10 (8)	6 (4)	1 (1)

Abbreviation: p, percentile

### Vitamin D intake reported pre-expansion in 2013–2014 and simulated post-expansion of the vitamin D fortification policy

Median (p25, p75) reported vitamin D intake from the VDQ among all participants was 3.8 µg/day (2.7, 5.3) in 2013–2014 prior to the vitamin D fortification expansion. The median simulated intake post-expansion was 7.4 µg/day (5.3, 9.9), which was statistically significantly different from the pre-expansion intakes (*p* < 0.001). Median change between reported vitamin D intake pre-expansion and simulated intake post-expansion was 3.5 µg/day (2.3, 4.7). Median vitamin D intake from fortified foods was 2.4 µg/day (1.4, 3.2) pre-expansion and 6.0 µg/day (3.8, 8.0) post-expansion. Total reported vitamin D intake from vitamin D-rich foods, and vitamin D intake from oily fish, margarine, milk, and fermented milk differed significantly by continents of origin, both pre- and post-expansion (*p* < 0.001 for all analyses) (Table 3). The highest total reported vitamin D intake (4.0 µg/day) pre-expansion was among participants born in Northern Europe and the largest simulated change (3.6 µg/day) between pre- and post-expansion was among participants born in Continental Europe. The lowest reported total vitamin D intake pre-expansion (3.0 µg/day) and lowest simulated change (2.9 µg/day) were among participants born in Asia. Post-hoc analyses of differences in reported intake of oily fish, margarine, milk, and fermented milk between continents of origin are found in Table 3.

**Table 3:** Reported daily intake of vitamin D from oily fish and fortified foods in 2013–2014 (pre-expansion) and simulated intake following the 2018 vitamin D fortification policy expansion (post-expansion), stratified by continent of origin

	Northern Europe (*N* = 1355)		Continental Europe (*N* = 131)		Asia (*N* = 173)		Africa (*N* = 102)		*p*-value^a^
	Median (p25, p75)	Mean rank	Median (p25, p75)	Mean rank	Median (p25, p75)	Mean rank	Median (p25, p75)	Mean rank	
**Oily fish**	1.8 (0.78, 1.82)	902^†^	0.8 (0.78. 1.82)	821	0.8 (0.26, 1.82)	758^†^	1.8 (0.78, 1.82)	884	0.001
**Margarine**									
Pre-expansion	0.6 (0.6, 2.0)	920^†^	0.6 (0.2, 2.0)	815^‡^	0.6 (0.2, 2.0)	662^†‡§^	0.6 (0.2, 2.0)	824^§^	< 0.001
Post-expansion	1.3 (1.3, 4.0)	920^†^	1.3 (0.5, 4.0)	815^‡^	1.3 (0.5, 4.0)	662^†‡§^	1.3 (0.5, 4.0)	824^§^	< 0.001
Change^b^	0.6 (0.6, 2.0)	920^†^	0.6 (0.2, 2.0)	815^‡^	0.6 (0.2, 2.0)	662^†‡§^	0.6 (0.2, 2.0)	824^§^	< 0.001
**Milk**									
Pre-expansion	1.0 (0.3, 1.0)	929^†‡§^	0.3 (0.0, 1.0)	758^†^	0.3 (0.0, 1.0)	654^‡^	0.5 (0.0, 1.0)	789^§^	< 0.001
Post-expansion	2.0 (0.5, 2.0)	901^†^	2.0 (0.5, 5.0)	877	2.0 (0.5, 2.0)	745^†^	2.0 (0.5, 2.0)	849	< 0.001
Change^b^	1.0 (0.3, 2.0)	886	1.0 (0.3, 2.5)	925	1.0 (0.3, 2.0)	804	1.0 (0.3, 2.0)	885	0.139
**Fermented Milk**									
Pre-expansion	0.2 (0.0, 0.8)	900^†^	0.0 (0.0, 0.4)	811	0.3 (0.0, 0.8)	887^‡^	0.0 (0.0, 0.2)	713^†‡^	< 0.001
Post-expansion	2.0 (0.5, 2.0)	917^†‡^	2.0 (0.5, 2.0)	842	0.5 (0.3, 2.0)	747^†^	0.5 (0.0, 2.0)	679^‡^	< 0.001
Change^b^	1.2 (0.4, 2.0)	916^†‡^	1.2 (0.3, 2.0)	869	0.5 (0.2, 1.2)	724^†^	0.5 (0.0, 1.3)	700^‡^	< 0.001
**Total intake** ^**c**^									
Pre-expansion	4.0 (2.8, 5.5)	926^†‡^	3.4 (2.1, 4.9)	752^†^	3.0 (1.5, 4.3)	651^‡§^	3.6 (2.2, 6.2)	839^§^	< 0.001
Post-expansion	7.8 (5.6, 10.1)	920^†^	6.8 (4.8, 9.8)	823^‡^	6.3 (3.1, 8.4)	650^c†‡§^	7.1 (4.3, 10.1)	825^§^	< 0.001
Change^b^	3.5 (2.5, 4.8)	910^†^	3.6 (2.2, 5.0)	905^‡^	2.9 (1.3, 4.2)	700^†‡^	3.0 (2.0, 4.5)	786	< 0.001

Abbreviation: p, percentile

Same footnote symbols (e.g., †, ‡, §) indicate statistically significant differences between continents of origin through post hoc analyses with Bonferroni correction

^a^ Kruskal Wallis test

^b^ Change in vitamin D intake calculated as the difference between post- and pre-expansion of the vitamin D fortification policy

^c^ Total intake of vitamin D from oily fish, margarine, milk, and fermented milk

**Fig. 2:**
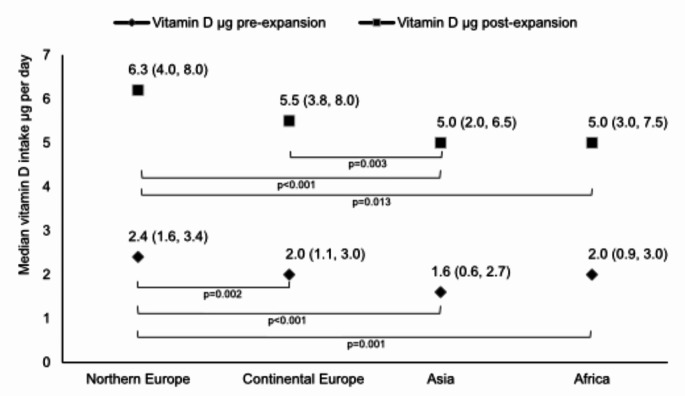
Median (percentile 25, percentile 75) reported vitamin D intake from fortified foods in 2013–2014 (pre-expansion) and simulated intake following the 2018 vitamin D fortification policy expansion (post-expansion), stratified by continent of origin. Bonferroni corrected p-values from pairwise comparisons between continents of origin

When the reported and simulated intakes of vitamin D were restricted to fortified foods, differences were also found between the continents of origin (Fig. 2). Median reported vitamin D intake in from fortified foods prior to the expanded vitamin D fortification policy was statistically significantly higher among participants born in Northern Europe (2.4 µg/day), compared to all other continents. Additionally, participants born in Northern Europe had a higher median simulated vitamin D intake from fortified foods post-expansion (6.3 µg/day), compared to Asia (5.0 µg/day, *p* < 0.001) and Africa (5.0 µg/day, *p* = 0.013)

### Reported frequency and amount of vitamin D-rich foods in 2013–2014

**Fig. 3:**
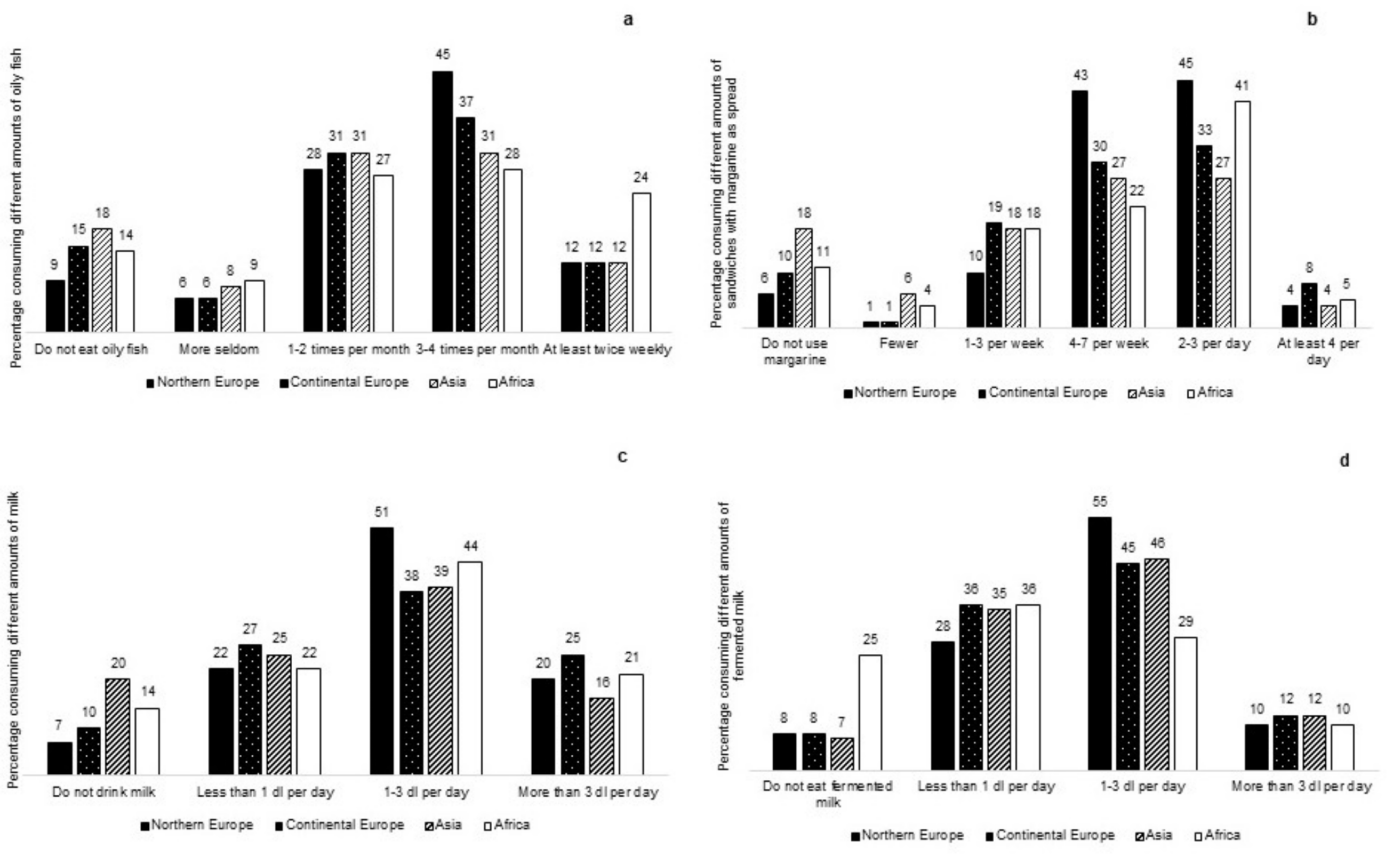
Percentage of participants who reported consuming different quantities of oily fish (**a**), margarine (**b**), milk (**c**), and fermented milk (**d**) in 2013–2014, categorized by continents of origin

Overall, a relatively high proportion of participants reported a high consumption of vitamin D-rich foods (Fig. 3a-d). However, the frequency of consumption for each food type varied depending on the continent of origin. For oily fish (Fig. 3a), the most commonly (28–45%) reported frequency among all participants was 3–4 times per month. Only 12% of all participants, with the exception of those born in Africa (24%), reported consuming oily fish at least twice weekly. Overall, participants from Northern Europe reported somewhat higher frequency categories for intake of fortified margarine, milk, and fermented milk (Fig. 3b-d) than participants from other continents. Of note, 18% and 20% of participants from Asia reported no intake of margarine and milk, respectively (Fig. 3b-c), while 25% of participants from Africa reported no intake of fermented milk (Fig. 3d).

### Reported types of milk and fermented milk consumption in 2013–2014

**Fig. 4:**
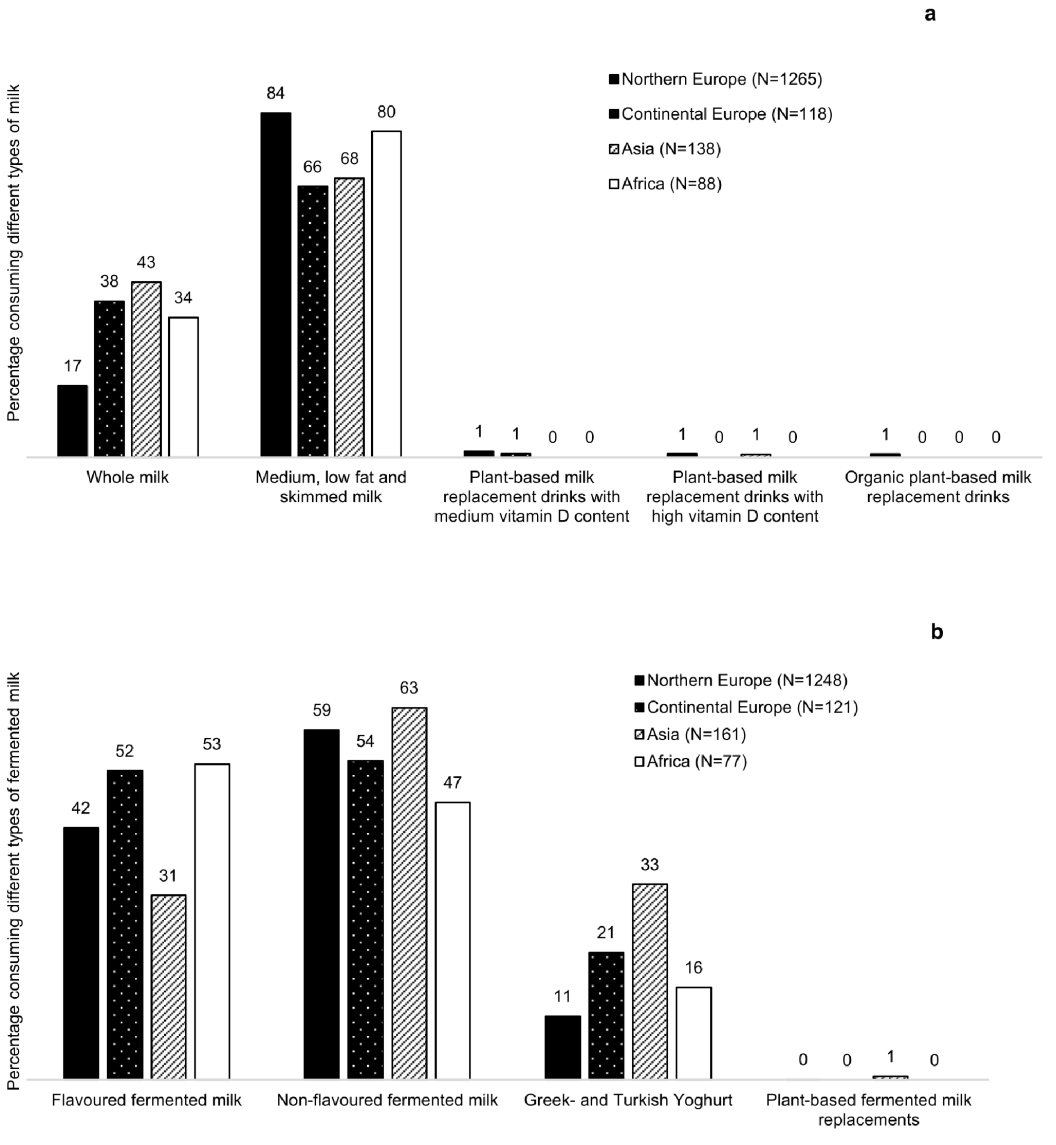
Percentage of participants who reported consuming different types of milk (**a**) and fermented milk (**b**) in 2013–2014 among milk and fermented milk consumers, categorized by continents of origin

Differences in the type of reported milk and fermented milk consumption were found between continents of origin (Fig. 4a-b). Overall, the most commonly reported types of milk were medium-, low fat or skimmed (66–84%). Whole milk consumption was reported by 43% of participants born in Asia, but only by 17% of those born in Northern Europe. Non-flavoured fermented milk was the most commonly reported type among all participants (54–63%), except for those born in Africa where flavoured fermented milk was the most common type (53%). Higher proportion of participants born in Asia reported consumption of Greek- or Turkish yoghurt (33%), compared to the other continents (11–21%). Consumption of plant-based milk and fermented milk alternatives were uncommon among all participants (0–1%).

## Discussion

To our knowledge, this is the first study to evaluate the potential impact of the extended fortification policy on vitamin D intake, among pregnant individuals in Sweden. The expansion of the Swedish vitamin D fortification policy appeared to benefit all participants; however, the potential impact of this expansion varied depending on the continent of origin. Specifically, the greatest potential benefit was observed among participants from both Northern and Continental Europe, as indicated by the highest simulated change in vitamin D intake between pre- and post-expansion. In contrast, the extended fortification policy had the least impact on vitamin D intake among participants from Asia and Africa.

The participants of Asian and African origin exhibited a lower consumption of fortified foods and a smaller simulated change in vitamin D intake between pre- and post-expansion, when compared to their Northern European counterparts. The lower reported intake of milk and fermented milk among participants of Asian and African origin compared to Northern Europeans partially explains this discrepancy. Additionally, participants of Asian origin had a higher reported intake of Greek- or Turkish yoghurt, which is not mandatory for fortification in Sweden [23]. Furthermore, they exhibited a higher proportion of participants who were non-milk and/or non-margarine consumers compared to participants from other continents of origin. Participants born in Continental Europe had the highest simulated change between pre- and post-expansion intake. This may be due to a higher proportion of participants from Continental Europe reporting the consumption of whole milk and flavoured fermented milk, now mandatorily fortified under the expanded policy [23, 24]. Previous research has indicated a lower consumption of dairy products among ethnic minority groups [28–30], but there is a lack of previous data during pregnancy in Sweden. A study conducted in Sweden found that non-pregnant women of African and Asian origin had lower intakes of vitamin D fortified margarine and milk compared to those born in Sweden [28]. However, a small Finnish study yielded contradictory results, indicating that the contribution of fortified fluid milk products to vitamin D intake was greater among African women than among Finnish women [31]. In that study, similar levels of vitamin D intake from fish were observed in these groups, which is consistent with our findings.

The lower reported consumption of food fortified with vitamin D among participants from Asia and Africa, compared to Northern Europeans, is concerning. This may pose a challenge to health equity, given the increased risk of insufficient 25-hydroxyvitamin D levels among of individuals of Asian and African origin [8, 25–27, 32]. Previous research suggests that ethnic minority residents with higher skin pigmentation at northern latitudes may have an increased vitamin D requirement due to limited sun exposure [33, 34]. Vitamin D supplementation has been demonstrated to contribute substantially to the intake of vitamin D during pregnancy in the Nordic countries [35–37]. However, a lower prevalence of vitamin D supplement use has been observed among individuals of African and Asian descent in early pregnancy [38]. It has been demonstrated that the effect of vitamin D supplementation in non-pregnant and pregnant individuals with obesity has a blunted response in 25OHD concentrations, suggesting that higher intakes of vitamin D may be needed in individuals with obesity [39, 40].

Unlike other Nordic countries, such as Finland, Iceland, and Denmark [13], there is no pregnancy-specific recommendation of vitamin D supplementation in Sweden. Instead, individuals who do not consume fish or fortified food, or have limited sun exposure are recommended to supplement with 10 µg of vitamin D daily [41]. The general Swedish dietary guidelines suggest a daily consumption of 5 dl of non-flavoured skimmed milk and low-fat fermented milk, and consumption of both oily and non-oily fish, 2–3 times weekly [42]. We found that only a minority of all participants reported consumption of more than 3 dl of milk and fermented milk per day, as well as oily fish at least twice weekly. Given this observation, it could be argued that the recommendation for vitamin D supplementation should be expanded to include individuals who consume fish and fortified foods in low amounts. This is particularly important in winter months at higher latitudes, where a consistent and higher intake of these foods becomes necessary to meet the recommended daily intake of vitamin D [12].

Due to the 2018 extension, the Swedish mandatory vitamin D fortification policy is now similar to the fortification policy in Finland. The Finnish policy, which has undergone prospective assessment, has resulted in improvements in vitamin D intake and status in the overall population including pregnant individuals [43, 44]. However, the Finnish evaluation did not present how ethnic minorities within the overall population benefitted from the changes. While vitamin D fortification of dairy products and margarine is a common strategy to increase vitamin D intake [13, 16–18, 23, 24], increasing the diversity of vitamin D fortified foods may be necessary to accommodate varying dietary habits across the entire population [45–47]. Various foods have been suggested as appropriate fortification vehicles e.g., flour, cooking oil, bread, breakfast cereals, and cheese [45–48]. A pertinent example is India, where vitamin D fortified cooking oil is included in the fortification programme [49]. Moreover, vitamin D fortified cooking oil has been demonstrated to be an effective strategy in improving vitamin D status among Iranian adults [50]. With regard to non-lipid-containing foods, wheat and maize flour have been identified as potential vehicles for micronutrient fortification [51]. These crops, along with rice, are important staple crops in East and Southeast Asia [52], yet there is a paucity of data on the consumption of such foods among individuals born in Asia and migrating to Sweden. Identifying appropriate vehicles for food fortification depends on a thorough understanding of the dietary patterns across the entire population [53]. Several aspects of fortification strategies, including coverage and bioavailability, must also be considered [45, 46, 54]. Further studies are needed to prospectively evaluate the impact of the expanded fortification policy in Sweden on the entire population, taking demographic minorities into account. To ensure accurate results, it is important to acknowledge potential differences between ethnic groups and avoid masking patterns in minority groups with different dietary habits [55].

The strengths of this study are its relatively large sample size and representativeness of the Swedish pregnant population in terms of origin, age, BMI, parity, and tobacco use [56], enhancing the external validity of the results. However, there are also some limitations of our study. Firstly, the VDQ utilized to evaluate vitamin D intake focused on only four distinct vitamin D food sources. Consequently, the VDQ failed to provide data on the complete dietary vitamin D intake. However, it is a validated tool for identifying individuals with high or low vitamin D intake [22]. Additionally, the VDQ captures the majority of vitamin D intake, and specifically the vitamin D fortified foods, identified as important contributors to the total vitamin D intake in the latest national survey of the general adult Swedish population [20]. Secondly, the data, collected 10 years ago, may not accurately reflect the current consumption of vitamin D fortified foods. Sweden introduced their expanded vitamin D fortification policy in 2018, with a two-year transitional period [23, 24]. Nevertheless, the expansion of the fortification policy was initiated as early as in autumn 2014 by some of the Swedish manufacturers of dairy products [57]. Consequently, to accurately estimate vitamin D intake from fortified foods prior to the implementation of the expanded vitamin D fortification policy, it is strengthened to study intakes prior to autumn 2014, as this study does. Thirdly, we can only simulate the impact of the expanded vitamin D fortification policy, and if vitamin D intake post-expansion differs by continent of origin. The simulation does not consider any changes in dietary intake that may have occurred since the data collection period in 2013–2014. However, by using the same study population to evaluate the impact of the expanded vitamin D fortification policy, we can estimate the direct potential effect of the expanded vitamin D fortification policy on simulated changes in vitamin D intake. In addition, dietary simulation models have previously been highlighted to provide valuable insights into policymaking and resource allocation [58]. However, comprehensive assessments of vitamin D intake and vitamin D status in contemporary cohorts would give important insights in how the current vitamin D fortification policy in Sweden supports vulnerable groups such as pregnant individuals, children, and the elderly.

## Conclusion

In conclusion, the simulated potential impact of the expanded Swedish vitamin D fortification policy benefitted vitamin D intake among pregnant participants of all origins. However, simulated change in vitamin D intake and intake from fortified foods post-expansion was lowest among participants born in Asia and Africa. This indicates that the current fortification policy is most advantageous for individuals of European origin, which may pose a challenge to health equity.

## Electronic supplementary material

Below is the link to the electronic supplementary material.

Supplementary Material 1

## Data Availability

The data that support this study are not openly available as they are subject to secrecy in accordance with the Swedish Public Access to Information and Secrecy Act [Offentlighets-och sekretesslagen, OSL,2009:400], but can be made available upon reasonable request. Data are in controlled storage at University of Gothenburg. Requests for data should be made to Hanna Augustin, hanna.augustin@gu.se.
