# Supplementary material for: The potential impact of the extended vitamin D fortification policy during pregnancy varies by continent of origin - a population-representative Swedish cohort
Source: Eur J Nutr. 2025 Jan 24;64(2):69. doi: 10.1007/s00394-025-03584-2 (PMC11861403; doi:10.1007/s00394-025-03584-2)
Supplement: Supplementary file 1 — Supplementary Material 1 [file 394_2025_3584_MOESM1_ESM.pdf]

Supplementary Information

The potential impact of the extended vitamin D fortification policy during pregnancy varies by continent of origin - a population-representative Swedish cohort

European Journal of Nutrition

Mathilda Forsby, Anna Winkvist, Ciara Mooney, Frida Dangardt, Jenny M Kindblom, Linnea Bärebring, Hanna Augustin

Corresponding Author

Mathilda Forsby

mathilda.forsby@gu.se

Department of Internal Medicine and Clinical Nutrition, Institute of Medicine, University of Gothenburg, Gothenburg, Sweden.

Do you eat oily fish (salmon, mackerel, herring)? ☐ NO ☐ YES

If YES: How often?

- ☐ At least twice weekly
- ☐ 3-4 times per month
- ☐ 1-2 times per month
- ☐ More seldom

Do you drink milk or is milk part of your diet? ☐ NO ☐ YES

If YES: How much milk do you consume?

- ☐ More than 3 dl (10 oz) per day
- ☐ 1-3 dl (3.5-10 oz) per day
- ☐ Less than 1 dl (3.5 oz) per day

If YES: Which type of milk do you consume most often?

- ☐ Whole milk
- ☐ Medium fat milk
- ☐ Low fat milk
- ☐ Skimmed milk
- ☐ Other (e.g., oat milk), please specify:…………………………………………………..

Is yoghurt and/or sour milk part of your diet? ☐ NO ☐ YES

If YES: How much yoghurt/sour milk do you consume?

- ☐ More than 3 dl (10 oz) per day
- ☐ 1-3 dl (3.5-10 oz) per day
- ☐ Less than 1 dl (3.5 oz) per day

If YES: What kind do you consume most often?

- ☐ Fruit-/vanilla yoghurt/sour milk
- ☐ Plain yoghurt/sour milk
- ☐ Turkish/Greek yoghurt

Do you use margarine as a spread (e.g., Bregott, Lätta, Milda, Becel)? ☐ NO ☐ YES

If YES: How many sandwiches with margarine do you eat?

- ☐ At least 4 per day
- ☐ 2-3 per day
- ☐ 4-7 per day
- ☐ 1-3 per week
- ☐ Fewer

Supplementary Fig S1. The short vitamin D questionnaire (VDQ).
